# Targeting Vascular Impairment, Neuroinflammation, and Oxidative Stress Dynamics with Whole-Body Cryotherapy in Multiple Sclerosis Treatment

**DOI:** 10.3390/ijms25073858

**Published:** 2024-03-29

**Authors:** Angela Dziedzic, Karina Maciak, Elżbieta Dorota Miller, Michał Starosta, Joanna Saluk

**Affiliations:** 1 Department of General Biochemistry, Faculty of Biology and Environmental Protection, University of Lodz, Pomorska 141/143, 90-236 Lodz, Poland; angela.dziedzic@biol.uni.lodz.pl (A.D.); karina.maciak@edu.uni.lodz.pl (K.M.); 2 Department of Neurological Rehabilitation, Medical University of Lodz, Milionowa 14, 93-113 Lodz, Poland; elzbieta.dorota.miller@umed.lodz.pl (E.D.M.); michal.starosta@umed.lodz.pl (M.S.)

**Keywords:** multiple sclerosis, neurodegeneration, neuroinflammation, vascular impairment, oxidative stress, whole-body cryotherapy

## Abstract

Multiple sclerosis (MS), traditionally perceived as a neurodegenerative disease, exhibits significant vascular alternations, including blood–brain barrier (BBB) disruption, which may predispose patients to increased cardiovascular risks. This vascular dysfunction is intricately linked with the infiltration of immune cells into the central nervous system (CNS), which plays a significant role in perpetuating neuroinflammation. Additionally, oxidative stress serves not only as a byproduct of inflammatory processes but also as an active contributor to neural damage. The synthesis of these multifaceted aspects highlights the importance of understanding their cumulative impact on MS progression. This review reveals that the triad of vascular damage, chronic inflammation, and oxidative imbalance may be considered interdependent processes that exacerbate each other, underscoring the need for holistic and multi-targeted therapeutic approaches in MS management. There is a necessity for reevaluating MS treatment strategies to encompass these overlapping pathologies, offering insights for future research and potential therapeutic interventions. Whole-body cryotherapy (WBCT) emerges as one of the potential avenues for holistic MS management approaches which may alleviate the triad of MS progression factors in multiple ways.

## 1. Introduction

Clinicians face significant challenges in understanding and treating conditions characterized by a complex, multifactorial, and elusive pathogenesis. A prime example may be multiple sclerosis (MS), an autoimmune and demyelinating disorder that affects the central nervous system (CNS) and is characterized by inflammation, loss of myelin sheaths, and ultimately neurodegeneration [1,2].

The MS pathogenesis is not clearly defined due to its highly varied and heterogeneous clinical and imaging manifestations [3]. Based on immanent features and symptom progression, two main clinical courses may be distinguished. The initially diagnosed relapsing–remitting stage (RR) is the most common form of MS characterized by periods of exacerbation (an acute inflammation relapsing) and followed episode resolutions (remission). The exacerbation usually occurs 1–2 times a year, for several days (from 24 h to one week), and then spontaneously reduces until it disappears, in the initial phase of the disease, but episodes worsen as it progresses [4]. The manifestation of RRMS symptoms during flare periods is the result of autoimmune inflammation in the CNS. Autoreactive immune cells, primarily T cells, are activated and cross the blood–brain barrier (BBB), leading to the destruction of myelin sheaths surrounding nerve fibers. However, inflammation is transient and remyelination occurs, and although it is not durable, neurological symptoms resolve completely in the initial phase of the disease [5]. Typically, as the disease develops, after an average of approximately 15 years since the first symptoms occur, approximately three-fourths of patients with RRMS advance into the secondary progressive stage (SP), which is marked by the progression of neurodegenerative phenomena leading to disability [4]. So, in the progressive subtype, during disease duration, the neurodegeneration processes are more prominent features than inflammation and gradually increase, leading to progressive deterioration of the patient’s disability. In addition to RRMS and SPMS, there is also primary progressive multiple sclerosis (PPMS), which is the rarest type of MS (observed only in approximately 10% of diagnosed individuals). The typical progression of symptoms seen in PPMS makes it appear similar to SPMS; however, in PPMS, the progressive disability begins at the very onset of the disease [6]. In progressive MS subtypes, over time, long-standing established astroglia activation, secretion of pro-inflammatory cytokines, chemokines, and free radicals cause persistent lesions in the gray and white matter of the brain and spinal cord. Demyelination and neurodegeneration in the CNS are associated with profound astroglia reactions, forming a dense glial scar in long-standing established inflammatory lesions. Focal demyelinated plaques diffuse and create large confluent demyelinated lesions. This includes deprivation of nerve fibers and permanent damage to axons and finally results in profound brain tissue loss and atrophy [7].

Many MS patients demonstrate increased susceptibility to cardiovascular diseases and impaired function of the BBB; such vascular damage not only indicates a pathology of the CNS, but also raises critical questions about the role of the BBB [8,9]. Such disruption allows inflammatory factors and immune cells to penetrate protected areas, such as the CNS, leading to a persistent state of inflammation, characterized by microglia activation and lymphocyte infiltration, thus contributing to tissue damage [10].

Moreover, neuroinflammation, marked by the accumulation and activation of immune cells within the CNS, serves as both response to and a driver of neurological damage; it plays a crucial role in the development of demyelinated plaques, whose presence is a distinctive feature of MS. Such a persistent inflammatory state poses a challenge to the clinician for distinguishing the protective responses from those that perpetuate disease progression [11].

Adding to this complexity is the role of oxidative stress, characterized by an imbalance between the generation of reactive oxygen species (ROS) and the capacity to neutralize them. In MS, oxidative stress is not merely a byproduct of neuroinflammation but a significant contributor to neuronal and axonal damage, which exacerbates the disease process [12].

**Figure 1:**
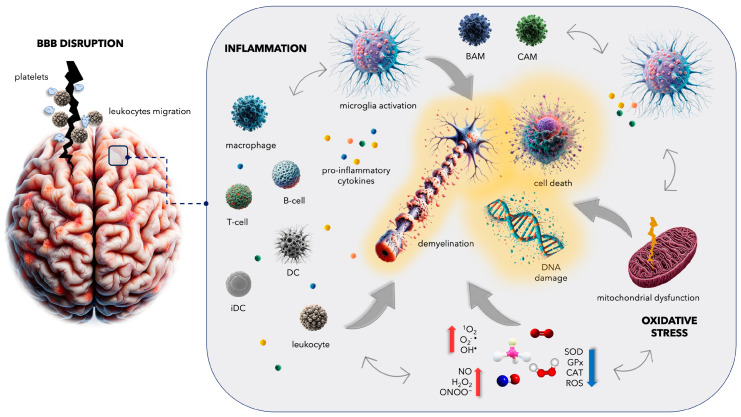
Multiple sclerosis (MS) manifests as inflammatory demyelination and neuronal death within the central nervous system (CNS), closely linked to blood vessel damage and heightened the permeability of the blood–brain barrier (BBB) [2]. The interaction between platelets and leukocytes entails an augmented production of pro-inflammatory cytokines and the recruitment of immune cells, including macrophages, dendritic cells (DCs), T cells, B cells, and immature monocyte-derived DCs (iDCs), traversing the disrupted BBB and initiating an inflammatory cascade. This process results in the persistent activation of microglia, collaborating with barrier-associated macrophages (BAMs) and CNS-associated macrophages (CAMs), and oxidative stress, thereby contributing to neuroinflammation and disease progression. Mitochondrial dysfunction, driven by increased reactive oxygen species (ROS), further amplifies neuronal damage [10,11,12]. Figure—own elaboration using elements generated by DALL·E 3, OpenAI.

Understanding the interplay between vascular damage, neuroinflammation, and oxidative stress is critical for comprehending the pathogenesis of neurodegenerative diseases. This triad of pathological processes not only coexist, but also actively contribute to the progression of existing disorders by reinforcing each other (Figure 1). To provide multifaceted therapeutic approaches for ameliorating disease progression and improving clinical outcomes, it is crucial to first understand the molecular basis of the key etiological components of the disease.

In neurological and psychiatric conditions, rehabilitation is typically focused on learning lost skills and becoming as independent as possible. Recently, many novel strategies in neurorehabilitation have been introduced, one of them being whole-body cryotherapy (WBCT), which is used in multiple sclerosis. WBCT and other cooling therapies are promising methods that can improve function and quality of life [13]. Although the neurobiological basis of WBCT in MS is not fully understood, it has been proposed that the procedure may decrease oxidative stress by enhancing antioxidative enzyme activity or partially inhibiting the generation of reactive oxygen species (ROS) [14]. Many studies have shown that exposure of the whole body to low temperatures can change the level of selected enzymes and hormones in bodily fluids.

Generally, evidence suggests that WBCT increases blood serum levels of adrenaline, noradrenaline, adrenocorticotropic hormone, cortisol, and testosterone and decreases certain parameters of inflammatory reactions [15]. However, no studies have yet assessed the effect of WBCT on molecular and morphological indices in patients with MS.

## 2. Methods

A search for peer-reviewed articles was conducted across multiple databases, including PubMed, Sage Journals, and SCOPUS, to compile relevant literature for this review. Additionally, Google Scholar was utilized to identify open-access articles. Data from authoritative sources such as clinicaltrials.gov were also incorporated into the analysis. The review encompasses 117 literature sources, comprising 64 original research studies (including case reports, clinical trials, and cohort studies) and 53 reviews (encompassing systemic reviews, literature reviews, and meta-analyses). The timeframe for the included publications ranges from 1994 to 2023, with a predominant concentration between 2019 and 2023. Identified relevant reviews underwent manual screening to ensure comprehensive coverage of pertinent references. Search terms utilized in this study encompassed a spectrum of topics relevant to multiple sclerosis, including neurodegeneration, neuroinflammation, vascular impairment, oxidative stress, and whole-body cryotherapy. Variations of these terms were employed to maximize the scope of search results. Boolean operators “AND” and “OR” were strategically applied after compiling all keywords, synonyms, and phrases to refine the search strategy and enhance result relevancy.

## 3. Complications in Multiple Sclerosis: From Blood–Brain Barrier Disruption to Increased Cardiovascular Disease Risk

Although MS is classified as an inflammatory demyelinating disorder of the CNS, it is intimately associated with vascular impairment, and with heightened permeability of the BBB [16]. Research has confirmed a higher susceptibility to cardiovascular diseases, notably ischemic stroke, myocardial infarction, and deep vein thrombosis, among patients with MS. Ischemic events are particularly closely linked to an intensified coagulation cascade and abnormal pro-thrombotic activity among platelets [17,18,19,20,21,22,23,24]. A population-based cohort study involving 17,418 Danish MS patients found that the disease itself to be a long-term risk factor for venous thromboembolism [25], while another analysis of 9881 MS patients found that they are over 30% more likely to die due to cardiovascular events than the general population [21]. A post-mortem analysis of 6000 Danish MS patients showed that the most common cause of death was not the disease itself, but cardiovascular diseases [24]. It has also been noted that the duration of the disease is one of the main risk factors for deep vein thrombosis [22].

A cohort study of 13,963 Danish MS patients found that the highest risk of stroke is within the first year after diagnosis and that it was most common among MS patients up to 56 years of age [22]; however, it is important to note that relapses occurring in the RRMS are often misdiagnosed as a stroke. Indeed, the opposite trend was observed in a Swedish cohort of 8281 MS patients which confirmed a positive correlation between age and risk of stroke [24]. In the later stages of the disease, the patient is typically not as fit, and a more sedentary lifestyle can elevate the risk of stroke [26]. Furthermore, stroke is strongly linked with deep venous thrombosis, the incidence of which increases by 40% in the late stage of MS [27]. Therapies for MS might also heighten the susceptibility to thromboembolic events, while disease-modifying therapies (DMTs) with anti-inflammatory properties could potentially mitigate cardiovascular risk factors [28].

It is, however, possible to reduce disease activity, influence the intensity of relapses, and treat concomitant clinical symptoms. Nevertheless, despite our more detailed knowledge about the pathomechanism of MS, the condition is characterized by a multitude of symptoms, and the possibilities for pharmacological treatment are unfortunately limited. Therefore, the key roles in treatment are played by physical activity and physiotherapy.

**Figure 2:**
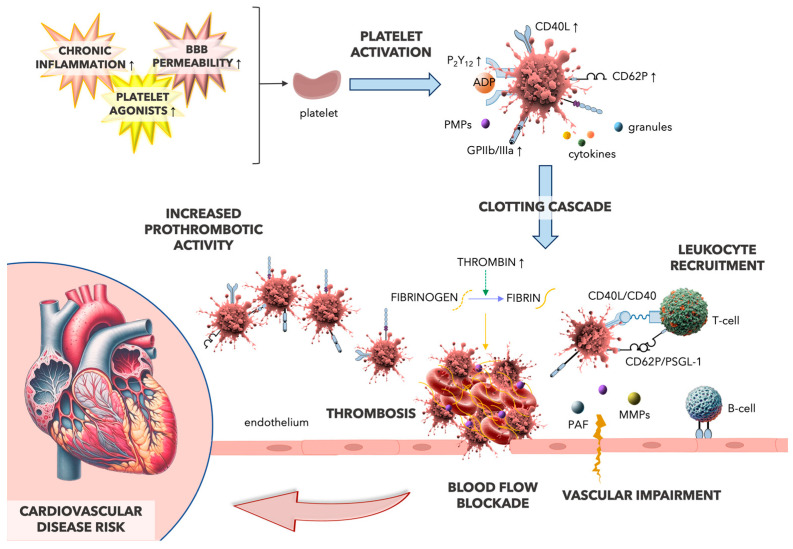
Multiple sclerosis (MS) pathophysiology includes chronic inflammation, blood–brain barrier (BBB) disruption, and the increased response to platelet agonists, leading to enhanced platelet activation and a predisposition towards pro-thrombotic phenotype and elevating the risk of cardiovascular disease [29]. In MS, platelets show increased expression of surface receptors, including P2RY12, CD40L, CD62P, and GPIIb/IIIa, which are essential for their activation. Once activated, platelets release molecules that trigger both pro-inflammatory and pro-thrombotic responses. The interaction between platelets, leukocytes, and endothelial cells, particularly through the CD40/CD40L and CD62P/P-selectin glycoprotein ligand (PSGL-1) pathways, exacerbates vascular impairment and activates the clotting cascade. The CD40/CD40L pathway, significant in autoimmune pathologies, enhances leukocyte adhesion and infiltration, leading to increased recruitment of immune cells to vascular injury sites [30,31,32,33]. Furthermore, the interaction between platelets and leukocytes intensifies vascular permeability involving platelet-derived microparticles (PMPs), platelet-activating factor (PAF), and matrix metalloproteinases (MMPs), disrupting endothelial junctions and promoting inflammation [30,31,32,33]. Figure—own elaboration using elements generated by DALL·E 3, OpenAI.

The increased risk of stroke, myocardial infarction, heart failure, and deep vein thrombosis observed in MS patients may all result from certain pathological mechanisms that are also implicated in cardiovascular diseases, such as oxidative stress, inflammation, and thrombotic factors (Figure 2) [29].

Pathological overactivation of blood platelets and thrombotic lesion formation within the wall of the coronary artery are widely recognized as major contributors to heart attack. Patients with acute myocardial infarction are characterized by elevated blood platelet activation and increased aggregation potential within the coronary circulation. Thrombotic occlusion of coronary vessels due to the formation of cellular aggregates can reduce or completely block blood flow [30]. Similarly, ischemic stroke can also result from the occlusion of cerebral blood vessels [31]. As the clotting cascade intensifies, excessive thrombin generation occurs; this enzyme converts soluble fibrinogen into insoluble fibrin, thus forming the clot responsible for venous thrombosis [32]. Thrombin serves as both the principal coagulation factor of the blood clotting cascade and the most potent agonist of blood platelets, thereby contributing to pro-thrombotic platelet activity [33]. However, in addition to thrombin, a number of endogenous physiological agonists are known to also activate blood platelets, with various consequences, including adhesion to the vessel wall, secretion of biologically active compounds accumulated in their numerous granules, and the generation of pro-thrombotic and pro-inflammatory microparticles. They can also increase receptor expression, which ultimately triggers the formation of platelet aggregates. The excessive activation of blood platelets is well known to play an important role in the pathophysiology of MS [34,35]. MS patients exhibit enhanced surface receptor activity and expression on blood platelets, as well as a heightened response to platelet agonists, such as adenosine diphosphate (ADP). They are also characterized by elevated mRNA expression of the *P2RY12* gene, in both blood platelets and megakaryocytes (platelets’ precursor cells), along with a higher density of P2Y12, a crucial ADP receptor, on the platelet surface. Platelets and megakaryocytes derived from patients with MS also demonstrate higher levels of fibrinogen and exhibit changes in the composition and genetic sequences of fibrinogen chains [36,37].

The inflammatory response causes plasma fibrinogen levels to rise two- to three-fold [38]. Fibrinogen can modulate the inflammatory response by activating leukocytes and synthesizing pro-inflammatory mediators [39]; it can also activate glial cells, resulting in BBB dysfunction in MS patients [40]. However, Ptaszek et al. [41] report no increase in fibrinogen levels in women with MS, although a significant increase was noted in healthy women after a series of 20 WBCT sessions.

Activated blood platelets play a pivotal role in the coagulation cascade by serving as primary contributors to cellular hemostasis. Additionally, they have also been implicated in the development of neuroinflammatory processes associated with MS. The activated coagulation cascade stimulates blood platelets, which in turn amplify coagulation [42]. Therefore, the thrombin cascade also plays a significant role in the inflammatory response in MS. Studies profiling the proteome specific to the cerebrospinal fluid (CSF) found that the coagulation cascade also plays a pivotal role in MS patients with an increased burden of cortical lesions [43]. Later studies found blood platelets to be ensnared in chronic active demyelinating MS lesions, thus confirming their participation in the pathogenesis of MS through involvement in the inflammatory reaction [44]. Most importantly, a decrease in the number of platelets together with blockage of the main surface receptor GPIIb/IIIa, determining pro-thrombotic activity, inhibited the inflammation process and markedly improved symptoms in experimental autoimmune encephalomyelitis (EAE) [45].

The inflammatory response is acknowledged to play a crucial role in neuronal disorders like MS. In MS in particular, chronic inflammation and heightened pro-oxidative activity are known to be primary factors inducing excessive blood platelet activation. Blood platelets interact with immune cells to exacerbate inflammation via various inflammatory and immune pathways.

Overactivated blood platelets have a strong influence on the inflammatory response, and this is directly correlated with their increased adhesiveness to altered endothelial cells or proteins within the subendothelial layer of blood vessel walls. Moreover, stimulation increases the ability of platelets to activate both leukocytes and dendritic cells and their tendency to form aggregates with leukocytes [46,47]. It is important to note that the platelets, immune cells, and endothelial cells are all activated by reciprocal cellular interactions taking place between them. This activation has serious implications for maintaining the integrity of the blood vessel wall and contributes to the escalation of the local inflammatory response.

The interaction between blood platelets and leukocytes generates platelet-activating factor (PAF), which increases BBB permeability by interrupting endothelial junctions [48,49,50]. Similarly, the formation of cellular aggregates increases the production of matrix metalloproteinases (MMPs) by leukocytes, which are generally recognized as significant participants in BBB disruption [51]. MMPs are also produced by overactivated blood platelets; these share platelet-derived microparticles (PMPs), which act as a reservoir of a range of biologically active proteins [52].

Another important determinant in autoimmune disease is the CD40/CD40L platelet–leukocyte inflammatory signaling pathway. Increased levels of sCD40L, the soluble form of CD40L, a membrane glycoprotein of the TNF family, have been found in serum and CSF from MS patients [53]. Cellular interactions mediate the adhesion of circulating leukocytes to the endothelium, facilitating their recruitment and initiating their diapedesis and infiltration into the inflamed vessel. This process enables the selective recruitment of leukocytes to inflamed areas of vascular wall injury.

One of the earliest phases of the creation of new demyelination lesions is endothelial monolayer dysfunction. Briefly, a pro-inflammatory and pro-thrombotic phenotype of the endothelium increases blood platelet activation and the adhesion and subsequent migration of immune cells to the subendothelial space, resulting in vasomotor alterations [54,55]. Assuming the hypothesis that the interplay between blood platelets, leukocytes, and endothelial cells contributes to the breakdown of the BBB, this may be the pivotal initial step in certain neurological inflammatory diseases, such as MS; this process leads to the infiltration of lymphocytes and subsequent formation of inflammatory lesions in the brain [56]. The autoimmune reaction initiates a significant influx of inflammatory cells and provokes pro-inflammatory activation of microglia cells. This cascade leads to the disruption of the myelin sheath, resulting in the formation of demyelinating lesions and subsequent axonal/neuronal degeneration.

A fundamental role in the initiation and propagation of demyelinating plaque formation is believed to be played by T lymphocytes. Autoreactive T cells infiltrate the CNS and subsequently release pro-inflammatory cytokines. These cytokines activate macrophages, triggering inflammation in the white matter and the subsequent destruction of myelin [42]. In peripheral blood, under physiological conditions, the ratio of T to B cells is 9:1; however, the size of the B-cell population increases during the progression of autoimmune disease [57]. Blood platelets primarily enhance T-cell adhesion to endothelial cells, thus contributing to the formation of lymphocyte–platelet conjugates, disrupting BBB permeability. Studies indicate that T cells exhibit greater adhesive properties compared to B cells. This may suggest that T lymphocytes are most prevalent in the areas with developing inflammation; however, the B cell population represents almost 40% of all lymphocytes infiltrating the CNS structures [58,59]. The intricate relationship between MS and cardiovascular events sets the stage for a deeper exploration of neuroinflammation as a fundamental factor in MS progression.

## 4. Inflammatory Mechanisms in Multiple Sclerosis: Insights into Microglial Activation and CNS Immune Responses

One of the primary pathological characteristics of MS is an inflammatory process marked by the accumulation of immune cells within the CNS. In general, MS patients experience both acute and chronic inflammation, and these inflammatory processes have a crucial impact on the clinical manifestations of the disease. The acute inflammation associated with relapses leads to sudden neurological symptoms, such as muscle weakness, sensory disturbances, and visual impairments. These symptoms typically resolve partially or fully between relapses, reflecting the temporary nature of the inflammation. In contrast, the chronic inflammation observed in progressive MS contributes to a gradual decline in neurological function, leading to increasing disability over time. The accumulated damage to myelin and axons impairs the transmission of nerve impulses, gradually diminishing the ability of the brain to control muscles, senses, and cognitive processes. The chronic stage of MS is characterized by the accumulation of microglia, specialized CNS immune cells, which remain activated and contribute to the ongoing degeneration of white and grey matter of the brain [60].

One significant aspect of MS pathology is the disruption of the BBB, which allows immune cells, primarily T and B lymphocytes, to infiltrate into the brain. This leads to the development of active demyelinated plaques, the hallmark of MS, visible on magnetic resonance imaging (MRI), characterized by damaged myelin, inflammation, edema, and destruction of axons and nerve fibers [61].

**Figure 3:**
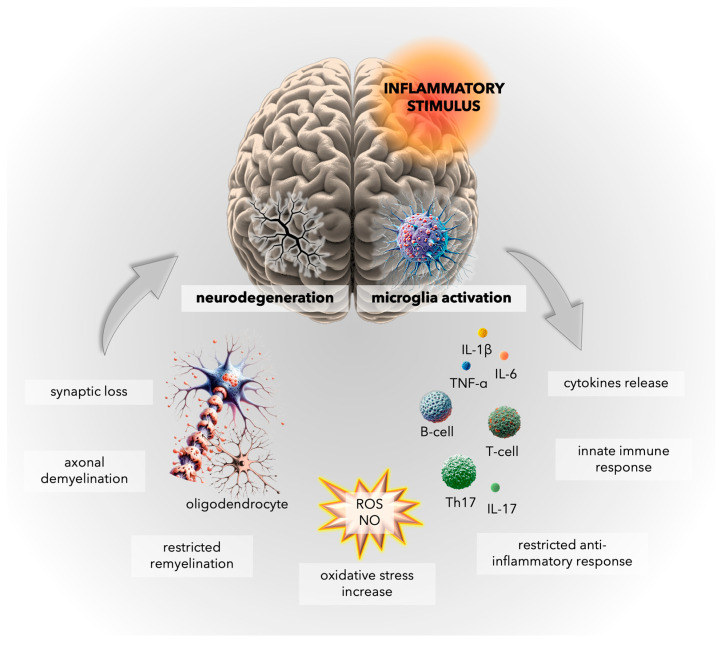
The balance between pro-inflammatory and anti-inflammatory activity of microglia is crucial for maintaining a balance between myelin loss and oligodendrocyte differentiation and remyelination. However, in the chronic stage of multiple sclerosis (MS), microglia accumulate and remain activated, contributing to white and grey matter degeneration [62]. The pro-inflammatory phenotype of microglia promotes the innate immune response, differentiation of T cells into T helper (Th)17 cells, generation of pro-inflammatory cytokines (e.g., tumor necrosis factor (TNF)-α, interleukin (IL)-1β, IL-6, IL-17), and reactive oxygen species (ROS) and nitric oxide (NO), all leading to synaptic loss and neurodegeneration [63,64,65,66,67]. Figure—own elaboration using elements generated by DALL·E 3, OpenAI.

Microglia play an essential role in maintaining homeostasis within the CNS. Under physiological conditions, microglia exhibit multiple, modulating functions in the brain, scavenging cellular debris, clearing apoptotic neurons, and promoting synapse formation. However, in response to injury, infection, or autoimmune processes, microglia generate an innate immune response and activate inflammatory mediators, exacerbating neuroinflammation and contributing to neurodegeneration (Figure 3) [62].

The inflammatory state in MS is characterized by both the pro-inflammatory and anti-inflammatory actions of monocyte-derived macrophages and microglia. Microglia are the innate immune cells native to the CNS, while monocyte-derived macrophages originate from peripheral blood monocytes and migrate into the CNS during inflammatory processes. Microglia can contribute to synaptic loss and cognitive decline by disrupting the homeostatic maintenance of neuronal synaptic plasticity [63]. Activated microglia and monocyte-derived macrophages intensify neuroinflammation by generating various pro-inflammatory cytokines, including tumor necrosis factor (TNF)-α and interleukins (IL)-1β and IL-6. These cytokines can also promote the differentiation of T cells into T helper (Th)17, further exacerbating the inflammatory response, and leading to neuronal damage and synaptic loss [64]. Nevertheless, the cells also play a vital role in tissue repair and recovery, clearing away debris and dead cells, and promoting oligodendrocyte differentiation and remyelination, which are essential for restoring myelin sheaths and restoring neural function [65,66]. Anti-inflammatory microglia express transforming growth factor (TGF)-β and cytokines such as IL-4 and IL-10, which can decrease the inflammatory response and promote tissue repair. The equilibrium between these two activations is crucial for preserving brain homeostasis [67].

In addition to microglia, barrier-associated macrophages (BAMs) and CNS dendritic cells (DCs) play critical roles in CNS immunity. Their ability to rapidly sense damage signals and modulate the immune response makes them crucial for maintaining CNS homeostasis and orchestrating the delicate balance between neuroinflammation and repair. During autoimmune-mediated neuroinflammation, the BBB becomes compromised, allowing immune cells from the peripheral circulation, including DCs, to enter the CNS [68]. BAMs interact with the BBB and monitor the constant molecular exchange between the bloodstream and the CNS; these are located in the non-parenchymal regions of the CNS, primarily in the meninges, choroid, and perivascular spaces, which all act as critical entry points for immune cells into the CNS [69,70]. BAMs actively participate in the neuroimmune response and communicate with astrocytes, the star-shaped glial cells that support neurons and contribute to maintaining the integrity of the BBB [71,72].

Early studies have shown that immature monocyte-derived DCs (iDCs) are more efficient at traversing the inflamed BBB than mature DCs. This is likely due to their ability to adhere more effectively to activated endothelial cells. Moreover, the adhesion between iDCs and BBB endothelial cells is mediated by adhesion molecules, including vascular (V)CAM-1, platelet endothelial (PE)CAM-1, intercellular adhesion molecule (ICAM)-1, and ICAM-2. While iDCs are more effective in passing the BBB, mature DCs are more selective in their crossing. Studies have shown that mature DCs are only affected by ICAM-1 [73]. This suggests that mature DCs require more specific signals to cross the BBB, perhaps indicating that they are only recruited in a severe state of CNS damage. CD18 and the DC-specific intracellular adhesion molecule-3-grabbing non-integrin (SIGN), expressed on both immature and mature DCs, also contribute to this process [74].

E- and P-selectins are adhesion molecules that play a crucial role in leukocyte recruitment during inflammation. However, it has been found that despite their elevated expression at the BBB during neuroinflammation, E- and P-selectins are not necessary for leukocyte recruitment across the BBB or the development of EAE [73,74]. Even so, it remains unclear whether selectins are essential for DC recruitment across the BBB, with current evidence being contradictory [75,76].

MS presents a multifaceted challenge for clinicians. It requires a comprehensive approach, balancing the need to prevent relapses, slow disease progression, effectively manage acute relapses and symptoms, and minimize the adverse effects of medications to manage the various aspects of the disease. Understanding the role of inflammation in MS has paved the way for the development of effective therapeutic strategies aimed at alleviating neuroinflammation, promoting tissue regeneration, and preventing neurodegeneration. Modern MS treatments aim to suppress the immune response and reduce the frequency and severity of relapses, slowing the progression of the disease and improving overall outcomes for patients.

The inflammation associated with MS may stimulate the respiratory burst system in activated microglia, raising ROS levels and thus increasing oxidative stress. ROS have been implicated as mediators of demyelination and axonal damage. It is assumed that the resulting free-radical-mediated tissue destruction may be prevented by antioxidants, which may also inhibit some of the early pro-inflammatory events and the trafficking of cells into the CNS [77]. It is hence recommended to support the mechanisms that can limit the severity of oxidative stress when treating diseases such as MS. One common approach is WBCT, which has been found to be very well tolerated by patients. A study of WBCT of MS patients by Bryczkowska et al. [78] found key antioxidant enzymes to act as reducing factors that neutralize the oxidative compounds before they can cause any damage to various biomolecules [79].

## 5. Oxidative Stress in Multiple Sclerosis: Unraveling the Complex Interplay

Persistent neuroinflammatory processes not only inflict direct damage to myelin sheaths and axonal structures but also create a milieu conducive to another critical aspect of MS pathology: oxidative stress. This alteration shifts the balance between the production of ROS and the effectiveness of endogenous antioxidant defense systems, thereby promoting neuronal damage and contributing to the progression of MS. Understanding the interaction between inflammation and oxidative damage is crucial for elucidating the pathophysiological underpinnings of MS.

### 5.1. General Facts about Oxidative Damage in MS

Oxidative stress is a key aspect of the pathophysiology of MS, resulting from a lack of balance between the production of ROS and the activation of antioxidant protection mechanisms, which is primarily influenced by the aberrant levels of the free radical scavenging enzymes [80]. Within the CNS, persistent inflammation and disrupted redox equilibrium impair brain plasticity, resulting in gradual demyelination and impaired neuronal signaling. These factors constitute the primary driver of psycho-motor disability. Oxidative stress is known to be associated with increased damage to myelin and axons in MS, and therefore, it almost certainly contributes to the observed clinical symptoms [81]. Furthermore, excess ROS production might trigger increased T-cell activation through an arachidonic acid cascade or inflict harm upon the BBB or myelin sheath, either directly or indirectly.

Despite the presence of natural antioxidant mechanisms, CNS cells, especially neurons and oligodendrocytes, are not entirely shielded against excessive ROS production. Indeed, the brain consumes 20% more oxygen than other organs, rendering it particularly susceptible to oxidative stress. Furthermore, brain cells are rich in polyunsaturated fatty acids with lower regenerative capacity [82], which additionally makes them more susceptible to oxidative damage.

Overproduction of highly reactive free radicals, including nitric oxide (NO), peroxynitrite (ONOO−), singlet oxygen (O_2_), superoxide anion (O_2_•−), hydrogen peroxide (H_2_O_2_), and hydroxyl radicals (OH•) [83], can lead to harmful protein oxidation, lipid peroxidation, nucleic acid damage, antioxidant enzyme inhibition, and programmed cell death pathway activation [84]. NO is a reactive nitrogen species (RNS) consisting of a free radical possessing an unpaired electron; it is a key regulator of blood flow and synaptic transmission [85]. Serum NO level is significantly higher in RRMS patients during relapse (*p* < 0.0001) [86]. The rapid reaction between NO and O_2_•− produces short-lived RNS, particularly highly reactive ONOO−, which may prompt oxidative/nitrative alterations in a diverse range of biomolecules [87]. Excessive NO production by immune cells induces hypoxia, hindering electron transport in the mitochondrial respiratory chain. This incomplete oxygen reduction triggers substantial ROS synthesis, altering mitochondrial structure and function and ultimately increasing oxidative stress [88]. Siotto et al. indicate elevated oxidative stress in RRMS patients with low levels of disability [89]. Moreover, clinical investigations have revealed increased oxidative stress in the bloodstream of patients with MS, characterized by dysregulated superoxide dismutase (SOD) [90] and glutathione (GSH) activity [91].

### 5.2. Mitochondrial Dysfunction in MS

Numerous human studies have provided compelling evidence indicative of mitochondrial dysfunction in patients with MS [92,93,94]. In mammalian cells, mitochondria represent a substantial source of ROS, and any impairment in the functionality of the respiratory chain could influence cell survival [95]. Persistent neuroinflammatory stimuli in MS disrupt neuroaxonal homeostasis, resulting in heightened oxidative stress marked by increased levels of ROS and mitochondrial damage. Such disruption increases excitotoxicity and disrupts the balance in neurotrophic factors needed to maintain neurons and oligodendrocytes [96]. The resultant impairment compromises mitochondrial functionality, exacerbating ROS generation and thus diminishing energy production efficiency. In this state, the cell is incapable of providing the necessary energy levels within demyelinated axons. As reduced ATP production reaches a critical point, ionic homeostasis becomes increasingly imbalanced, triggering the activation of apoptosis mechanisms [97,98].

Although ROS can damage mitochondria by altering their protein and lipid content, their predominant impact is observed on mitochondrial DNA, where they inactivate promoters and suppress mitochondrial gene expression. It is assumed that increased generation of ROS in mitochondria, encompassing species with prolonged half-life, such as H_2_O_2_ or lipid hydroperoxide, particularly may precipitate mitochondrial dysfunction, disrupting biological processes and contributing to various metabolic diseases [99].

Furthermore, previous studies suggest a reduction in peroxisome proliferator-activated receptor-gamma coactivator (PGC)-1α levels, a transcriptional factor crucial for regulating mitochondrial function; this decline is notably evident in the pyramidal neurons in SPMS and PPMS. This reduction in PGC-1α was correlated with the diminished expression of key components in mitochondrial machinery, such as oxidative phosphorylation (OXPHOS) subunits and antioxidants. These findings were confirmed with a functional model employing neuronal cells, which demonstrated a correlation between these alterations and increased ROS production [100].

Moreover, an independent study found that elevated ROS levels adversely impact the binding capacity of nuclear factor erythroid 2-related factor (NRF)-2, a transcription factor governing electron transport chain (ETC) proteins in SPMS patients, even within seemingly normal regions of the gray matter cortex [101]. In progressive MS patients (including 14 SPMS and 5 PPMS patients), heightened ROS production in the CNS was linked to an increase in the number of mitochondria in axons and astrocytes. Higher levels of ROS have been correlated with the translation of mitochondrial proteins in both active and inactive MS lesions; this is evidenced by heightened expression of proteins originating from the mitochondrial ETC complex IV and elevated levels of the mitochondrial stress marker mtHSP70 compared to controls [100].

A study comparing 10 post-mortem brains from individuals with MS (including 9 with SPMS and 1 with PPMS) revealed distinct alterations in the MS cortex in comparison to the control group. Notable differences in both nuclear DNA and mitochondrial (mt)DNA transcripts were observed between the groups. Furthermore, functional analysis conducted on identical samples revealed diminished activity in complexes I and III from neurons localized in the motor cortex of patients with MS; this was accompanied by a decline in GABAergic synaptic elements [102]. A separate study involving 13 SPMS patients identified significant mtDNA deletions in neurons, with some of these mutations being specific to the subunits of complex IV [103].

### 5.3. Genetic Factors Potentially Responsible for Oxidative Stress in MS

There has been a growing interest in the genetic predisposition to generating free radicals and the occurrence of MS. Evidence suggests that genetic factors, including single-nucleotide polymorphisms (SNPs) in genes encoding enzymes involved in oxidative stress pathways, play a pivotal role in determining treatment outcomes for MS.

The *NOX3* gene, located on chromosome 6q25.1, is responsible for synthesizing NADPH oxidase 3 (NOX3), an enzyme primarily found in the plasma membrane of the lung and cerebral cortex. NOX3 facilitates the generation of O_2_•− through a single-electron reduction of O_2_, with NADPH serving as the electron donor [104]. NOX3-generated ROS are particularly common in oligodendrocyte precursor cells, where they influence their differentiation and sensitivity to oxidative stress. It has been proposed that NOX3 generation stimulates oligodendrocyte maturation in MS patients [105]. Carlström et al. [86] report a significant association between the G allele of the rs6919626 (G > A,T) polymorphism in the *NOX3* gene and a decrease in ROS production by monocytes following ex vivo stimulation (*p* = 0.057) in a study of 564 RRMS patients in Sweden. This allele was also linked to a diminished response to dimethyl fumarate [106].

The *GSTP1* gene, located on chromosome 11 in the q13.2 region, is responsible for encoding the glutathione S-transferase P protein, an antioxidative enzyme that facilitates the merging of endogenous GSH. This enzyme is implicated in the modulation of MS progression, as disease advancement appears correlated with oxidative stress resulting from inflammation [107]. One extensively investigated polymorphism associated with varied responses to natalizumab is rs1695 (A > G, Ile105Val) [108]. A study on the rs1695 polymorphism found an improvement in disability among RRMS patients harboring the A allele (χ^2^ = 0.031; df = 1; *p* = 0.861) [108].

The *NQO1* gene, located on chromosome 16 in region q22.1, is responsible for producing an antioxidative enzyme known as NAD(P)H quinone oxidoreductase (NOQO1). Its function is to catalyze the double-electron reduction of quinones, thereby impeding their involvement in the redox cycle and ROS production [109]. One study found the rs1800566 polymorphism (C > T, Pro187Ser) to correlate with the reaction to natalizumab in RRMS patients; the identified association was particularly noticeable in MS patients possessing the C allele (χ^2^ = 3.320; df = 1; *p* = 0.068) [108].

WBCT offers promise for treating various disorders. WBCT is currently being used to relieve symptoms in diseases affecting the musculoskeletal system and the nervous system, such as MS. The application of low-temperature stimuli to the entire body is known to influence the autonomic, endocrine, circulatory, neuromuscular, and immunological systems [110]. Indeed, Capodaglio et al. [111] report that WBCT is an effective anti-inflammatory and antioxidant treatment that minimizes secondary tissue damage by reducing the production of pro-inflammatory and oxidative substances.

Attempts should be made to rehabilitate patients with MS throughout the course of the disease, not only during hospitalization. Such rehabilitation should be multidisciplinary and address both motor function and mental status. The rehabilitation protocol itself should be individualized and include physical rehabilitation intended to reduce spasticity and improve balance and posture [112]. Many studies underline the need for continued research into the effectiveness of different physiotherapeutic methods and types of therapies; however, current studies indicate that an effective approach is based on a combination of pharmacological treatment and physiotherapy.

It is known that ROS play an important role in demyelination [41], and hence, oxidative stress is believed to be one of the key factors involved in the pathogenesis of MS. Indeed, patients with MS show tend to present higher oxidative stress indices, as well as greater DNA failure [113]. Studies indicate that a course of at least 10 WBCT sessions can improve antioxidant potential: although MS patients usually demonstrate higher oxidative stress than healthy subjects, this value decreases after WBCT. While further research is clearly needed, WBCT appears to be an effective method of improving the antioxidant capacity of the body [114].

## 6. Whole-Body Cryotherapy (WBCT): Antioxidant Effects and Clinical Implications

The treatment approaches for MS include pharmacological interventions and various methods of physical medicine and rehabilitation. The latest research highlights the importance of exercise as a key therapy that can improve the functional status of MS patients. However, about 60–80% of MS patients are heat-sensitive, and exposure to a warm environment could potentially result in a transient exacerbation of symptoms. Therefore, WBCT and cooling therapies are very promising methods that can reduce fatigue and improve functional status and quality of life [13].

Recent clinical investigations indicate that WBCT has an antioxidant effect in MS patients resulting in improvements in plasma total antioxidative status, SOD, and uric acid levels [41,115]. However, there is a need to conduct more extensive clinical trials involving larger cohorts of participants and with consistent protocols. In Poland, WBCT is widely applied; according to National Health Fund data, 14,239 of the total 641,737 covered cryochamber therapy services were performed each year with MS patients during 2010–2019, representing about 2.3–2.5% of the total.

The molecular mechanisms responsible for the effectiveness of cryotherapy in minimizing oxidative damage remain unclear. Nevertheless, some studies have indicated that the induction of hypothermia may play a role in partially inhibiting ROS generation [14].

It has been proposed that long-term WBCT decreases oxidative stress by enhancing antioxidative enzyme activity. Repeated exposure to acute cold temperatures over several months may induce adaptive mechanisms, potentially contributing to body hardening. This adaptation to cold stimuli is believed to enhance protection against oxidative stress [116].

Siems et al. [117] discovered that regular winter swimming or intense endurance exercise increased enzymatic protection, suggesting activation of the antioxidant defense. Our studies showed that MS patients treated with three cycles of 10 exposures in a cryogenic chamber increased total antioxidant status (TAS) (an indicator of redox status) in plasma and improved disease symptoms. Moreover, SOD and CAT activities were further enhanced during WBCT sessions using melatonin supplementation (10 mg daily), without affecting TAS levels. The combined approach showed promise, emphasizing the need for further research on the antioxidative mechanisms of WBCT in MS treatment [96].

## 7. Conclusions

Our analysis confirms that the progression of MS is characterized by a critical convergence of vascular impairment, neuroinflammation, and oxidative stress, which form an intricate interplay. It is important to consider that MS manifests not as a singularly defined neurological disorder but rather as a spectrum of intertwined pathological processes.

A pivotal factor in the pathophysiology of MS is vascular impairment, particularly the disruption of the BBB. This disruption facilitates the infiltration of autoreactive immune cells in the CNS, thereby setting the stage for sustained neuroinflammation. The resulting inflammatory milieu, characterized by activated microglia and infiltrating lymphocytes, contributes to the demyelination and degeneration of the axons that are pathological features of MS. The pro-inflammatory factors released in this environment further perpetuate BBB permeability, creating a vicious cycle of inflammation and vascular compromise.

A critical role is also played by oxidative stress, which serves as both a consequence and a driver of neuroinflammation and vascular damage. Excessive production of ROS in this milieu causes direct damage to cellular elements, including DNA, proteins, and lipids. This oxidative damage not only exacerbates demyelination but also impairs remyelination processes, crucial for CNS repair. Mitochondrial dysfunction, particularly in neuronal cells, underscores the significance of oxidative injury in MS. The complex interrelation between oxidative stress and inflammatory and vascular pathology represents a multifaced aspect of MS pathogenesis.

This understanding calls for a holistic approach to the management and treatment of the disease, where therapeutic strategies should be multi-targeted and personalized, addressing the specific needs and pathophysiological profiles of individual patients. Incorporating modalities such as WBCT, which involves the application of extremely cold temperatures to affected areas, can aid in reducing inflammation and promoting vascular health. Additionally, WBCT can complement rehabilitative efforts for individuals recovering from a stroke, helping to improve motor function and overall well-being. Thus, the integration of WBCT into holistic therapies and rehabilitation programs for MS patients can offer promising avenues for comprehensive care and management of the condition.

## Data Availability

No new data were created or analyzed in this study. Data sharing is not applicable to this article.
